# Multimodal Body Representation of Obese Children and Adolescents before and after Weight-Loss Treatment in Comparison to Normal-Weight Children

**DOI:** 10.1371/journal.pone.0166826

**Published:** 2016-11-22

**Authors:** Simone Claire Mölbert, Helene Sauer, Dirk Dammann, Stephan Zipfel, Martin Teufel, Florian Junne, Paul Enck, Katrin Elisabeth Giel, Isabelle Mack

**Affiliations:** 1 Department of Psychosomatic Medicine and Psychotherapy, University Medical Hospital, Tübingen, Germany; 2 Max Planck Institute for Biological Cybernetics, Tübingen, Germany; 3 Graduate Training Centre of Neuroscience, International Max Planck Research School, University Tübingen, Tübingen, Germany; 4 Fachkliniken Wangen i.A., Children Rehabilitation Hospital for Respiratory Diseases, Allergies and Psychosomatics, Wangen i.A., Germany; Charité-Universitätsmedizin Berlin, Campus Benjamin Franklin, GERMANY

## Abstract

**Objective:**

The aim of the study was to investigate whether obese children and adolescents have a disturbed body representation as compared to normal-weight participants matched for age and gender and whether their body representation changes in the course of an inpatient weight-reduction program.

**Methods:**

Sixty obese (OBE) and 27 normal-weight (NW) children and adolescents (age: 9–17) were assessed for body representation using a multi-method approach. Therefore, we assessed body size estimation, tactile size estimation, heartbeat detection accuracy, and attitudes towards one’s own body. OBE were examined upon admission and before discharge of an inpatient weight-reduction program. NW served as cross-sectional control group.

**Results:**

Body size estimation and heartbeat detection accuracy were similar in OBE and NW. OBE overestimated sizes in tactile size estimation and were more dissatisfied with their body as compared to NW. In OBE but not in NW, several measures of body size estimation correlated with negative body evaluation. After weight-loss treatment, OBE had improved in heartbeat detection accuracy and were less dissatisfied with their body. None of the assessed variables predicted weight-loss success.

**Conclusions:**

Although OBE children and adolescents generally perceived their body size and internal status of the body accurately, weight reduction improved their heartbeat detection accuracy and body dissatisfaction.

## Introduction

Childhood obesity is increasing worldwide, and it is associated with both psychosocial and medical complications [1, 2]. Awareness of the problem and motivation are considered a key factor in changing health behavior [3, 4]. In this sense, it has been suggested that a lack of awareness of the own body size or indifference towards own weight status contribute to overweight, as they hamper motivation for weight loss [5–7]. In addition, it has been suggested that a disturbed interoceptive processing, as indicated by poor heartbeat detection accuracy, might contribute to an excessive food intake [8, 9]. As yet, no study has comprehensively investigated different types of body representation in obese children and adolescents. It is still unclear whether obese children and adolescents really have a disturbed body representation and whether weight loss also involves changes in body representation that could be addressed in weight loss treatment.

Body representation is not uniform, but a conglomerate of multiple body representations that are informed by different modalities [10, 11]. In this notion, body representation comprises not only attitudes about body weight and shape, but also a mental picture of one’s own body and implicit representations informed by proprioception, somatosensation and interoception. It is assumed that different body representations are organized along a continuum between implicit and explicit representations [12].

Studies on childhood obesity have typically focused on explicit body representation only and mostly used cross-sectional designs. It has been shown that a significant proportion of overweight children tend to underestimate their current body size in figure rating scales [13, 14], though this has not been replicated in adults when using methods that have a less social focus [15]. Also, children and adolescents with a high body mass index (BMI; kg/m^2^) tend to have high body dissatisfaction and low self-esteem, contradicting the idea that the problem of obesity is often ignored [1, 13, 16]. Longitudinal studies, suggested that both body dissatisfaction and body size estimation in figure rating tasks approach performance of normal-weight children when overweight is reduced [17–19].

Recently, more implicit types of body representation came into the focus of obesity research and reopened the debate again. Heartbeat detection accuracy has been observed to be diminished in adults with high BMI [20] and it is associated with healthier eating behavior and better physical fitness in children [9, 21]. Also, studies in adults indicate that participants with high BMI might have difficulties in estimating the size of an object touching their skin (tactile size estimation), possibly reflecting a disturbed sense of the own size [22–24]. Taken together, while the observed disturbances in explicit measures of body representation could be interpreted as effect of social teasing and stigmatization, implicit measures suggested that an inaccurate representation of the own body size could still play a role in obesity.

In the present study, we wanted to obtain a more comprehensive picture of a possible body image disturbance in childhood obesity than previously reported. Specifically, we aimed to find out i) whether and in which measures of body representation obese children and adolescents differ from normal-weight mates matched for age and gender and heartbeat detection ii) how the different measures of body representation are associated with each other in both groups. In addition, we followed up obese children and adolescents until discharge from a weight-loss therapy, as we wanted to investigate iii) whether weight loss induced any changes in the body representation of obese children and adolescents. Finally, we also wanted to test iv) whether any of the body representation measures would serve as a predictor for weight loss success in obese children, as suggested by health behavior theories [3, 4].

## Materials and Methods

### Study design and Participants

The study presented here was conducted as part of the DROMLIN-study (PreDictor Research in Obesity during Medical care—weight Loss in children and adolescents during an INpatient rehabilitation) [25]. The study protocol was approved by the Ethics Committee of the medical faculty for the University Tübingen, Germany. This study is registered at the German Clinical Trials Register (DRKS) with the clinical trial number DRKS00005122.

Children and parents were informed about the study purpose and provided written consent prior to inclusion. In short, 60 overweight and obese children (OBE; age 9–17, 47% male) with a BMI over the 90^th^ percentile for their age and sex specific norms [26] and an indication for hospitalization for weight loss intervention were included. All OBE participated in a weight loss program at the Children Rehabilitation Hospital for Respiratory Diseases, Allergies and Psychosomatics in Wangen i.A., Germany. The program comprised physical activity, cognitive behavioral therapy, and a balanced diet. A detailed description of the setting is reported elsewhere [25]. Exclusion criteria were severe psychological comorbidities, linguistic or intellectual limitations, type-1 diabetes, malignant tumors, systemic disorders, or severe cardiovascular diseases. Additionally, 27 normal weight children (NW; 11–14 years, 56% male) matched for age and gender with a BMI between the 10^th^ to 90^th^ BMI percentile from the surrounding area of the University Hospital Tübingen, Germany were recruited and served as control group.

OBE were tested twice, upon admission (T1) and prior to discharge (T2). The anthropometric and body perception assessments took place in an afternoon session and the heartbeat perception assessment in the morning session. NW were tested once in a single session, and served as control group for T1.

### Assessments

#### Anthropometry

The physical development of the children was assessed using the tanner stages [27, 28]. The tanner scale ranges between 1 (prepubertal) and 4 (mature). In the context of anthropometric measurements, the actual body widths (spine, hip, thigh, upper arm) and body depths (abdomen, buttocks), were measured with a caliper and body circumferences (abdomen, buttocks, thigh, upper arm) with a tape measure, respectively in the morning. Participants were not informed about their body dimensions.

#### Body size estimation

Two hours after the anthropometry, the same investigator assessed the corresponding body size estimations by instructing the participants to set their dimensions by moving sliders on a 2 m wooden slat. Then, the investigator measured the adjusted dimension without providing any feedback. At the beginning of each trial, the investigator placed both sliders in the middle of the slat. The children’s cognitive ability to discriminate between physical dimensions was tested by presenting everyday objects of different size that had to be estimated: a mobile phone (9 cm), a book (24 cm), and a bottle (34 cm). After each presentation, the object was removed and the participant was asked to set its length on the wooden slat.

#### Tactile size estimation

We conducted a tactile size estimation test similar to the one reported by Keizer et al. [29] at four different body sites (upper spine, upper arm, buttocks, thigh). The participants were blindfolded and the investigator pressed a small caliper/pair of compasses with predefined distances on different body sites. After each tactile stimulation (each distance and body site), the blindfold was removed and the participants had to reproduce the perceived distance using the wooden slat. The distances between the two points were as follows: spine– 20 cm, upper arm– 10 cm, buttocks– 15 cm, thigh– 10 cm.

#### Perception indices and scores

A perception index for each body size and tactile size estimate was calculated according to the formula: perception index = (estimated / actual body size) x 100 [30]. Next, mean perception scores for each group were calculated as average of the single measures for everyday objects (mobile phone, book, bottle), body width (spine, hip, thigh, upper arm), body depth (abdomen, buttocks), body circumference (abdomen, buttocks, thigh, upper arm) and tactile size estimation (spine, upper arm, buttocks, thigh). Values below 100 indicate an underestimation and values above 100 indicate an overestimation in terms of percent of the actual size.

#### Heartbeat detection

The heartbeat detection task was performed as reported previously by Pollatos and Schandry [31] in a modified version. During the procedure, a conventional ECG (3991/3-GPP BioLog, UFI Company, Morro Bay, CA) recorded the actual cardiac activity while the child was comfortably seated in a chair and was not allowed to speak and to move. A short test interval of 15 seconds was followed by four intervals of 25, 35, 45 and 55 seconds. Between the intervals were resting periods of 30 seconds. The children were instructed to count during each interval their own heartbeats by concentrating on their heart activity. The procedure was standardized by giving the instructions from a tape. A heartbeat detection index for every interval was calculated by the following formula: 1-(|recorded heartbeats–counted heartbeats|/recorded heartbeats). Next, the mean heartbeat detection score was calculated as average of the heartbeat detection indices of the four intervals 25s, 35s, 45s and 55s. The maximum score is 1, the minimum score is 0. A high index or score indicates a good concordance between the detected and actual heartbeat whereas a low score indicates a poor agreement between the detected and the actual heartbeat.

#### Concerns about body weight and shape

Eating behavior and concerns about body weight and shape were assessed with the validated Eating Behaviour and Weight Problems Inventory for Children (EWI-C), consisting of 60 items and 10 subscales [32]. In this study, the subscales “figure dissatisfaction” (consisting of 5 items), and “concerns about eating” (consisting of 8 items) are reported. Percentile ranks for the values of the subscales were retrieved by sex and age specific norm tables. Values between the 16^th^ and 84^th^ percentile can be considered as normal.

### Statistical analyses

The data were analyzed using SPSS version 19. Normally distributed data are presented as mean±standard deviation. Non-normally distributed data are presented as median [interquartile range] and the perception indices additionally by mean±standard deviation. Differences between OBE T1 and NW were calculated using unpaired t-tests (age, weight, height, BMI-SDS), Chi^2^ test (sex) or Mann-Whitney-U-tests if data were not normally distributed (EWI-C, perception indices). Differences between OBE T1 and OBE T2 were analyzed with paired t-test (weight, BMI-SDS) or Wilcoxon signed-rank test if data were not normally distributed (EWI-C, perception indices). We used Spearman correlations to analyze associations between variables, because in all analyzed pairs, at least one variable was not normally distributed. In order to analyze the association between body representation distortion and successful weight loss in OBE, Spearman correlations between the T1 perception scores and the delta BMI-SDS were calculated. The same Spearman correlations were computed using the T1 absolute values of mis-estimation instead of the T1 perception scores. Spearman correlations were computed for correlation analyses between all perception scores and EWI-subscales. In order to control for multiple testing the p-values were false discovery rate (FDR) adjusted [33]. FDR-values of <0.05 and for correlation analyses <0.15 were considered as statistically significant.

## Results

Table 1 provides an overview on the characteristics of the study population. At T2, seven children had dropped out so that the longitudinal data refers to a sample of 53 obese children. The length of intervention in OBE was 38±10 (min-max: 16–70) days. To exclude possible age effects, all analyses were repeated excluding the four youngest children (aged 9 to 10 years from the OBE group), which however, did not influence the results. Similarly, we explored whether results would change if absolute values of percentage of mis-estimation instead of perception scores were used. Again, this was not the case.

**Table 1:** Characteristics of the study population

				p-value	p-value
	OBE T1 (n = 60)	OBE T2 (n = 53)	NW (n = 27)	OBE T1 vs. OBE T2	OBE T1 vs. NW
Sex (♂:♀)	28:32	23:30	15:12	n.a.	n.s.
Age (years)	13.03±1.89	13.04±1.85	12.5±0.9	n.a.	n.s.
[Min-Max]	[9–17]	[9–17]	[11–14]		
Weight (kg)	84.0±20.5	80.9 ± 19.9	45.4 ± 8.2	**<0.001**	**<0.001**
[Min-Max]	[51.0–132.0]	[47–128]	[33.8–63.0]		
Height (cm)	163.1±10.5	163.3±9.9	158.1±9.4	n.s.	n.s.
[Min-Max]	[140–185]	[140–185]	[141–174]		
BMI-SDS	2.51±0.6	2.3±0.6	-0.2±0.6	**<0.001**	**<0.001**
[Min-Max]	[1.1–3.7]	[0.6–3.6]	[-1.3–1.1]		
EWI-C Figure dissatisfaction	83[68–93]	86[72–97]	35[28–42]	n.s.	**<0.001**
EWI-C Concerns about eating	90[78–96]	84[78–90]	25[24–53]	**0.005**	**<0.001**

Characteristics of obese children (OBE) before (T1) and after weight loss (T2) and the normal-weight children (NW) are displayed along with subscales of the validated Eating Behaviour and Weight Problems Inventory for Children (EWI-C [32]). Normally distributed data are presented as mean±standard deviation and non-normally distributed data as median[interquartile range]. Data were compared between OBE T1 versus OBE T2 and OBE T1 versus NW, respectively. P-values <0.05 were considered as statistically significant. Min = Minimum, Max = Maximum, n.a. = not applicable, n.s. = not significant.

### Group differences OBE T1 versus NW

**Fig 1:**
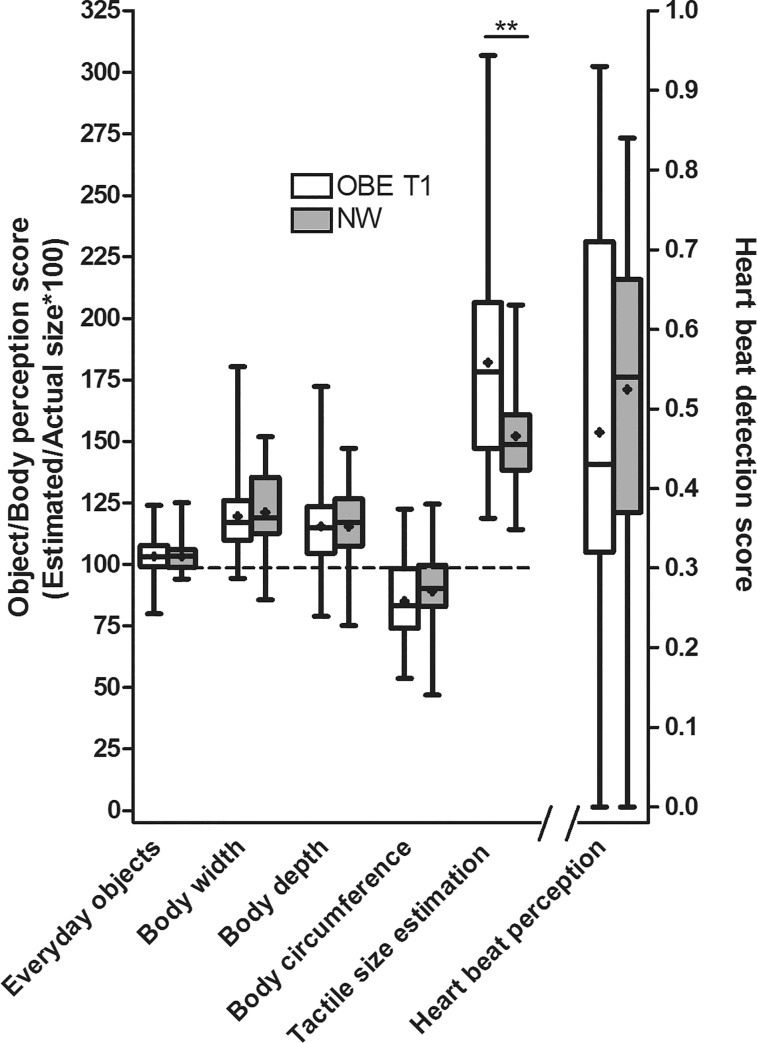
Perception Scores of body perception in obese children (OBE) before weight loss (T1) in comparison to normal-weight children (NW). The perception scores of everyday objects, body width, body depth, body circumference, tactile size estimation and heartbeat detection are displayed as box-whiskers with a cross, the latter depicting the mean. Except for the heartbeat detection score, values below 100 indicate an underestimation and values above 100 indicate an overestimation in terms of percent of the actual size. For the heartbeat detection, a score of 1 indicates absolute accuracy of heartbeat detection and the minimum score of 0 indicates that no heartbeat was perceived. The mean±standard deviation of the perception scores were as follows: *Everyday objects*–OBE: 103±08, NW: 103±07; *Body width*–OBE: 120±15, NW: 121±17; *Body depth*–OBE 115±20, NW: 115±17; *Body circumference*–OBE: 85±16, NW: 89±16; *Tactile size estimation*–OBE: 182±41, NW: 152±25; *Heartbeat detection*–OBE: 0.47±0.26, NW: 0.52±0.20. Due to multiple testing the p-values were false discovery rate (FDR)-adjusted. FDR values <0.05 were considered as statistically significant. ** indicates FDR<0.01.

As displayed in Table 2, both groups overestimated their body widths and body depths while they underestimated their body circumferences. However, the perception indices of body widths, body depths and body circumferences for the different body sites did not differ significantly between OBE and NW. As a result, the corresponding three aggregated perception scores “Body widths”, “Body depths” and “Body circumferences” were also similar in OBE versus NW (Fig 1) respectively. Both groups greatly overestimated the distances in the tactile size estimation task (Table 2). However, the perception indices “Spine”, “Buttocks” and “Thigh” of this task differed significantly between OBE and NW, with OBE overestimating the distances more than NW (Spine: U(N = 87) = 419.5, FDR = 0.005); Buttocks: U(N = 87) = 498.5, FDR = 0.04; Thigh: U(N = 87) = 342.5, FDR<0.001; Table 2). Consequently, the aggregated perception score “Tactile Size Estimation” also differed between OBE and NW (U(N = 87) = 434.0, FDR = 0.007, d = 0.81; Fig 1). In order to exclude that either changes in mechanoreceptor density through growth or central nervous system maturation influenced tactile size estimation performance, we explored whether performance correlated with the children’s height and their tanner stages, which was not the case. The heartbeat detection indices for the different counting intervals were similar in OBE and NW in all intervals (Table 2) and consequently also the aggregated detection accuracy score (Fig 1). The results for the two subscales “Figure dissatisfaction” and “Concerns about eating” are presented in Table 1. In both subscales OBE scored significantly higher than NW (“Concerns about eating”: U(N = 87) = 40.00, p<0.001, “Figure dissatisfaction”: U(N = 87) = 72.00, p<0.001), reflecting higher body dissatisfaction and eating concern in obese children.

**Table 2:** Perception indices of body perception in obese children (OBE) before (T1) and after weight loss (T2) in comparison to normal-weight children (NW).

	Mean±SD			Median [IQR]			FDR	FDR
Perception indices	OBE T1	OBE T2	NW	OBE T1	OBE T2	NW	OBE T1 vs. OBE T2	OBE T1 vs. NW
**Body widths**								
Spine	105±17	102±17	103±18	105[93–116]	101[90–111]	99[90–113]	0.4576	0.6554
Hip	114±16	114±16	108±19	114[103–124]	111[103–121]	106[96–117]	0.9823	0.1652
Thigh	126±24	130±15	137±43	124[107–140]	130[118–140]	130[109–164]	0.3822	0.471
Upper arm	133±38	135±29	137±28	127[109–155]	131[111–152]	141[123–151	0.6547	0.471
**Body depths**								
Abdomen	124±27	128±19	126±25	122[108–141]	127[117–142]	130[108–151]	0.6512	0.8016
Buttocks	107±21	110±18	104±21	103[93–120]	107[95–124]	105[90–117]	0.3822	0.9102
**Body circumferences**								
Abdomen	80±23	77±17	87±19	77[68–88]	76[64–88]	82[72–101]	0.3822	0.302
Buttocks	75±17	70±15	75±17	73[64–90]	69[58–80]	74[64–89]	0.1257	0.9102
Thigh	93±23	95±21	91±26	90[77–113]	96[77–108]	97[69–111]	0.8624	0.9102
Upper arm	92±27	98±22	102±23	92[73–108]	96[82–109]	100[89–113]	0.3822	0.3112
**Tactile size estimation**								
Spine	178±45	162±31	145±29	170[146–199]	156[144–171]	150[119–166]	0.1257	***0*.*0049***
Upper arm	180±76	196±63	181±57	170[127–228]	190[140–238]	169[139–206]	0.1257	0.9102
Buttocks	176±49	167±40	149±25	171[143–210]	163[139–197]	140[130–169]	0.6513	***0*.*0378***
Thigh	194±76	205±64	133±34	184[143–236]	100[159–252]	138[108–155]	0.131	***0*.*0005***

Perception indices of everyday objects, body widths, body depths, body circumferences, tactile size estimations and heartbeat detection accuracy are presented as mean±standard deviation and as median[interquartile range] due to the non-normal distribution of most data. Except for the heartbeat detection indices, values below 100 indicate an underestimation and values above 100 indicate an overestimation in terms of percent of the actual size. For the heartbeat detection, a score of 1 indicates absolute accuracy of heartbeat detection and the minimum score of 0 indicates that no heartbeat was perceived. The perception indices were compared between OBE T1 versus OBE T2 and OBE T1 versus NW, respectively. Due to multiple testing the p-values were false discovery rate (FDR)-adjusted. FDR values <0.05 were considered as statistically significant.

### Correlations between body representation measures

Correlations between all the perception scores and the subscales of the EWI-C were computed separately for OBE (at T1) and NW (Table 3). In OBE, the perception score “body width” correlated weakly to moderately with the perception scores “body depths”, “tactile size estimation and the EWI-C scale “eating concern”. The perception score “body depth” correlated weakly with “tactile size estimation”. A moderate correlation was observed between the two EWI-C scales “eating concern” and “figure”.

**Table 3:** Correlations between perception scores and subscales of the Eating Behaviour and Weight Problems Inventory for Children (EWI-C)[32] in OBE and NW group.

	Body width	Body depth	Body circumference	Tactile size	Heartbeat	EWI-C EC	EWI-C FD
**Body width**		0.42	-0.40	*0*.55**	-0.05	-0.02	-0.08
**Body depth**	*0*.41**		0.21	0.28	-0.05	0.05	-0.17
**Body circumference**	0.17	0.09		-0.18	0.17	0.21	0.02
**Tactile size estimation**	*0*.27*	*0*.26*	-0.21		-0.14	0.05	0.01
**Heartbeat detection accuracy**	-0.05	0.12	-0.09	-0.11		0.02	0.2
**EWI-C Eating Concern**	*0*.31*	0.03	*0*.29*	0.09	-0.19		*0*.75**
**EWI-C Figure Dissatisfaction**	-0.1	-0.17	0.04	-0.21	-0.25	*0*.43**	

Spearman correlations were computed and the correlation coefficients rho are presented for obese children (OBE, white background) and normal-weight children (NW, grey background). The p-values were false discovery rate (FDR) adjusted for multiple testing. A FDR <0.15 was considered as statistically significant.

* indicates FDR <0.15 but ≥0.05

** indicates FDR<0.05.

In NW, the perception score “body width” correlated moderately with “tactile size estimation”. Further, the perception score “body width” correlated with “body depth” and “body circumference”, but this finding did not withstand FDR-adjustment. In NW, the two EWI-C scales “eating concern” and “figure” correlated strongly with each other. Neither in OBE nor in NW, correlations between the heartbeat detection score and other perception scores or EWI-C scales were found.

### Changes in OBE body representation between T1 and T2

**Fig 2:**
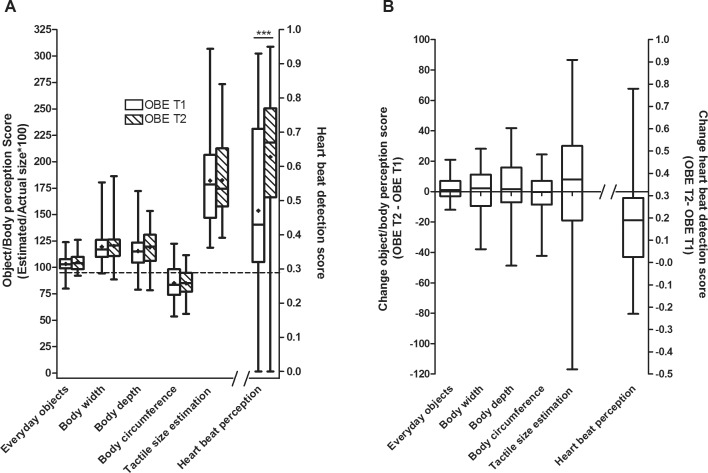
Perception Scores of body perception in obese children (OBE) before (T1) and after weight loss (T2). **A:** The perception scores of everyday objects, body width, body depth, body circumference, tactile size estimation and heartbeat detection are displayed as box-whiskers with a cross, the latter depicting the mean. Except for the heartbeat detection score, values below 100 indicate an underestimation and values above 100 indicate an overestimation in terms of percent of the actual size. For the heartbeat detection, a score of 1 indicates absolute accuracy of heartbeat detection and the minimum score of 0 indicates that no heartbeat was perceived. The mean±standard deviation of the perception scores were as follows: *Everyday objects*–T1: 103±08, T2: 105±08; *Body width*–T1: 120±15, T2: 120±14; *Body depth*–T1: 115±20, T2: 119±16; *Body circumference*–T1: 85±16, T2: 85±12; *Tactile size estimation*–T1: 182±41, T2: 183±35; *Heartbeat detection–*T1: 0.47±0.26, T2: 0.63±0.21. Due to multiple testing the p-values were false discovery rate (FDR)-adjusted. FDR values <0.05 were considered as statistically significant. *** indicates FDR<0.001. **B:** The change values of the perception scores in OBE are presented as box-whiskers.

In the OBE group, the weight loss treatment induced neither a significant effect with regard to any of the aggregated perception scores for “Body widths”, “Body depths”, Body circumferences” nor for the “Tactile Size Estimation” (Fig 2A). We also analyzed the individual changes in aggregated perception scores between T1 and T2 and found no trend of improvement or worsening (Fig 2B). In contrast, the heartbeat detection accuracy improved significantly in the course of weight loss from T1 to T2 in all examined intervals (Table 2) and in the aggregated score (Z(N = 52) = -5.174, FDR<0.001, d = 0.67, Fig 2B). Also, we observed that OBE improved in the EWI-C subscale “Concerns about eating” (Z(N = 53) = -2.81, p = 0.005).

### Prediction of weight loss success

None of the assessed measures of body representation at T1 correlated with the weight loss success (delta BMI-SDS).

## Discussion

Our observations suggest that obese children and adolescents generally represent their bodies as accurate as normal weight age mates, though in OBE, body size representation was associated with eating concern. Our observation that none of the assessed variables predicted weight loss success is contradictory to ideas that a lack of awareness of their excess body size or poor interoception contributes to being overweight. However, we observed that in the obese children and adolescents, not only eating concern, but also heartbeat detection accuracy improved throughout weight loss, suggesting that the program induced improvements in interoceptive processing.

We observed no uniform group differences between OBE and NW with regard to their general body size perception and heartbeat detection accuracy, but only in tactile size estimation and body dissatisfaction. In the body size estimation task, our observations do not confirm previous results from figure rating tasks suggesting that obese children underestimate their size [13, 14, 34]. Rather, our observations match findings obtained with depictive methods in adults by [15] that showed no difference between obese and normal-weight participants. The discrepancy may be due to the fact that figure rating tasks assess own body size perception as compared to a certain social range, whereas metric body size estimation, as used in this this study, assesses body size estimation for the actual size. Several studies have already shown that families and peers of obese children often do not perceive the child as obese, which may be beneficial for the child’s quality of life [35–37]. It is likely that obese children do not see themselves as different from their peers as they are, and thus underestimate in figure rating tasks while they might be accurate in tasks that do not require a social comparison.

Our observation that OBE children were less accurate than NW children in tactile size estimation, is in line with previous findings in adults [24]. Interestingly, differing from these previous studies, we found that both groups overestimated, but OBE children did so to a significantly higher degree. Tactile size estimation performance did neither correlate with height nor with tanner stages of physical development. We therefore consider it unlikely that differences in growth and maturation processes might have caused the group differences, as previous studies suggested [38, 39]. Rather, our results are similar to those found in anorexia nervosa suggesting that tactile size, despite being considered to assess implicit body representation, might be influenced top-down, e.g. by body dissatisfaction [24]. Hence, it might be the case that the overestimation of OBE children reflects their perception of being large although correlations between body dissatisfaction and tactile size estimation did not yield significance.

Heartbeat detection accuracy is assumed to be the central construct underpinning other interoceptive measures [40]. Further, it has been observed to be negatively correlated with the tendency to evaluate one’s body based on appearance rather than for its effectiveness [41]. Our observation of no group differences in heartbeat detection accuracy contradicts previous claims that a diminished perception of the inner status of the body might contribute to overweight [20]. Interestingly, in a large sample of children (n>1500) aged 6 to 11 years, no differences were observed between overweight and normal-weight children at a first assessment, whereas differences between the groups were evident one year later [9]. Our observations suggest that diminished heartbeat perception is likely not a general symptom of obesity. However, heartbeat perception is involved in weight regulation, possibly as a mediator for body-related cognitions.

Finally, our observation of high body dissatisfaction in OBE children confirm Wardle and Cooke [1],who identified high body dissatisfaction in OBE children as one of the main factors of their compromised psychological well-being. At the same time, our observation suggests that it is not the case that obese children have a lack of awareness of the problem but that they are aware of and suffering from their excess weight.

Group wise correlation analyses of the different measures of body representation revealed an interesting pattern: Whereas in the NW group questionnaire measures of eating concern and body dissatisfaction were independent from other body representations, high eating concern was associated with body size overestimation in the OBE group. This indicates that in obese children and adolescents, cognitions of being too fat are possibly internalized to a higher degree than previously assumed and thereby might influence body size estimation on a very basic level.

Interestingly, we found that different measures of body representation do not homogeneously correlate with each other. In both the OBE and the NW group, measures related to size estimations were correlated moderately with each other, but not with heartbeat detection accuracy. This supports the notion of body representation as conglomerate of multiple rather independent representations and emphasizes the necessity of a multi-method account.

Our third research question asked for the role of body representation in weight loss treatment. Although, heartbeat detection accuracy is unlikely to be involved in the etiology of overweight, our observation of improved heartbeat detection accuracy at T2 indicates that weight loss treatment affects interoception. From our data, it is unclear whether heartbeat perception accuracy is rather a marker or even a potential moderator variable for weight loss. A possible mechanism of this relationship includes physical fitness, which has been observed to be associated with heartbeat detection accuracy in high BMI children [21]. Alternatively, it could reflect changes in body image, as weight loss might reduce tendencies to evaluate oneself based on appearance [41]. However, the causal structure of this association is yet unknown.

In line with other studies, we also observed that body dissatisfaction, as reflected by the scale “eating concern” improved throughout the weight loss treatment [17, 18, 42, 43]. It has to be noted that body dissatisfaction remained on a level higher than in the NW group even at the end of the program. However, this suggests that positive effects on psychological well-being apply as soon as weight loss starts.

With regard to the predictive power of body representation for weight loss success, we found that none of the investigated measures predicted weight loss success of OBE children. Opposed to widely used models of health behavior change, our results suggest that a lack of awareness and, consequently, motivation for weight loss, is not the main hurdle for weight loss in obesity [3, 7, 44]. However, we observed body dissatisfaction to be very sensitive to weight loss, suggesting that motivational variables might be relevant for therapy adherence and success.

It is a limiting factor of this study i) that we were not able to analyze body representation in longer follow-up intervals and ii) that our design does not allow us to disentangle whether the observed changes in body dissatisfaction and interoception were rather consequences or even actively contributing to weight regulation. Although we have not found an immediate link between weight and body representation, it is still possible that some of the body representations investigated here are associated with weight loss, weight loss maintenance or weight gain in a long-term perspective. Studies with a longer follow-up interval and more measurement time intervals could help to clarify this question and to learn more about the mechanisms through which body representation and weight regulation interact.

There are also several strengths to our study. To our knowledge, we are the first to examine body representation of obese children from a multi-modal perspective and in both a cross sectional and a longitudinal setting. That way, we were not only able to compare body representation of obese children to normal weight children, but could also identify changes that occur in the course of weight loss. We observed that obese children do not have general problems to represent their excess body size. However, correlation analyses indicate that their self-categorization as “too large” is likely influencing their body representation on a basal level. Further studies focusing on the association between perception and representation of the body might help to better explain this observation.

For clinical practice, it is important that we observed counter-evidence for the idea that obese children lack awareness of their excess size or motivation to lose weight. Still, we observed that interoceptive awareness, as indicated by heartbeat detection accuracy, changes throughout weight loss therapy, suggesting that it might play a role in weight regulation. Further research is needed that tracks different types of body representation throughout development and long-term treatment of overweight. Specifically, the role of interoceptive awareness for weight loss treatment needs further exploration. Our findings also show that neither high body dissatisfaction nor accurate awareness of the own excess size translate into higher weight loss success. However, to improve psychosocial well-being of overweight children, weight loss interventions that specifically target body image may be useful.
